# Diabetic peripheral neuropathy in type 2 diabetes: prevalence and associated factors in Midwest Brazil

**DOI:** 10.20945/2359-4292-2026-0053

**Published:** 2026-05-17

**Authors:** Lorrany Campos de Queiroz Melo, Gleyson Souza da Costa, Matheus Eduardo Soares, Adriana Marilia Zanato Teruel, Augusto Baumgart de Liz, Josiane Neves Coelho Marques, Arthur Emílio Leite de Figueiredo, Luiz F. Viola, João Gabriel Guimarães Luz, Adriana Santi

**Affiliations:** 1 Programa de Pós-graduação em Biociências e Saúde, Universidade Federal de Rondonópolis, Rondonópolis, MT, Brasil; 2 Curso de Medicina, Universidade Federal de Rondonópolis, Rondonópolis, MT, Brasil; 3 Centro de Diabetes e Endocrinologia de Rondonópolis, Rondonópolis, MT, Brasil

**Keywords:** Type 2 diabetes mellitus, peripheral neuropathy, prevalence, risk factors, cardiovascular disease

## Abstract

**Objective:**

This study investigated the prevalence and factors associated with diabetic peripheral neuropathy (DPN) among adults with type 2 diabetes mellitus (T2DM) attending a referral center in Midwest Brazil.

**Subjects and methods:**

This cross-sectional study included 276 patients with T2DM, categorized into DPN and non-DPN groups. DPN was assessed using the Neuropathy Disability Score. Sociodemographic and lifestyle data were collected via structured interviews, whereas clinical and laboratory data were obtained from medical records.

**Results:**

DPN prevalence was 64.5% (95% CI: 58.9%–70.1%). Among those affected, most were female (59.0%), aged over 67 years old (37.7%), living with diabetes for more than 10 years (52.3%). Poor glycemic control and a sedentary lifestyle were observed in 78.9% and 85.4% of participants, respectively. In multivariable analysis, male sex (PR = 1.32; 95% CI: 1.12–1.56), retirement/inactivity (PR = 1.43; 95% CI: 1.11–1.85), T2DM duration >10 years (PR = 1.22; 95% CI: 1.03–1.44), sedentary lifestyle (PR = 1.34; 95% CI: 1.02–1.76), peripheral arterial disease (PR = 1.25; 95% CI: 1.08–1.46), and prior acute myocardial infarction (PR = 1.21; 95% CI: 1.01–1.45) were independently associated with higher DPN prevalence.

**Conclusion:**

DPN was highly prevalent among patients with T2DM. Particular attention should be directed toward men, older adults, individuals with long-standing diabetes, and those with cardiovascular comorbidities. Promoting regular physical activity and comprehensive management of cardiovascular risk factors may help prevent disease progression and related complications.

## INTRODUCTION

Type 2 diabetes mellitus (T2DM) is a chronic disease of high global prevalence, affecting approximately 828 million people in 2022, with a diagnostic frequency of 10.2% in the Brazilian population ^(1)^. Diabetic neuropathy is the most prevalent microvascular complications, affecting more than 50% of patients over the course of the disease, commonly presenting as diabetic peripheral neuropathy (DPN) ^(2)^.

Progressive degeneration of sensory neurons predisposes individuals to multiple adverse outcomes that negatively impact their quality of life, including pain-related physical disability, ulcers, infections, and lower-limb amputations. These complications can also lead to mood disorders, sleep impairment, and reduced work performance, resulting in a significant psychological, social, and physical burden ^(3,4)^.

DPN prevalence vary widely, from 1% to 80% across countries, reflecting differences in diagnostic criteria, assessment methods, patient age, diabetes duration, and presence of comorbidities ^(3-6)^. Studies show that Africa and South and Central America have some of the highest DPN prevalence. Associated risk factors include advanced age (> 70 years), longer duration of diabetes, poor glycemic control, metabolic syndrome (obesity, hypertension, low HDL-c, hypertriglyceridemia), sedentary lifestyle, smoking, and alcohol consumption ^(4-8)^. Importantly, DPN can be prevented or delayed by adequate glycemic control and lifestyle changes targeting modifiable risk factors ^(2,9)^.

In Midwest Brazil, few studies have evaluated the prevalence and factors associated with this complication at secondary care settings. Dutra and cols. ^(10)^ investigated risk factors for foot ulceration among 117 patients with diabetes treated in outpatient units of three public hospitals in the Federal District; however, foot ulcers represent a DPN progression. Similarly, Reis and cols. ^(11)^ evaluated predictive factors for DPN in a small sample of 111 older patients followed at a primary health unit in the same state.

Muzy and cols. ^(12)^ conducted a comprehensive analysis to assess the prevalence of diabetes and its complications, and the characteristics of diabetes care in Brazil, using data from three national surveys: the National Survey of Health, the Primary Care Access and Quality Improvement Program, and the Popular Pharmacy Program. The authors found that neuropathy was the most prevalent diabetes-related complication nationwide, that only 30% of patients underwent foot examinations, and that the Midwest had the second-highest neuropathy incidence (3,114.7 cases per 100,000 inhabitants), second only to the Southeast (3,496.0 per 100,000).

Despite these findings, none of these studies directly examined the factors associated with DPN, even though identifying such determinants is crucial to improve preventive strategies and optimize disease management within public health systems ^(13)^. Accordingly, this study investigated the prevalence and associated factors of DPN among individuals with T2DM treated at a referral center in Midwest Brazil.

## SUBJECTS AND METHODS

This cross-sectional study involved a sample of patients receiving care at a referral health service in the municipality of Rondonópolis, Mato Grosso, Midwestern Brazil, between August 2021 and December 2023. Sample size was estimated with Epi Info software, version 7.2.6.0 (Centers for Disease Control and Prevention, Atlanta, GA, USA), considering a population size of 1,464 individuals, corresponding to the annual number of patients with T2DM assisted at the studied referral health service. A 5% significance level, a 95% confidence interval (95% CI), and an expected 31.5% DPN prevalence were adopted, based on a meta-analysis by Sun and cols. ^(14)^ conducted with individuals with T2DM. These parameters resulted in a minimum required sample size of 270 participants.

Participants were invited to join the study while in the waiting room prior to their appointments. Adult patients (≥ 18 years), both males and females, with a diagnosis of T2DM confirmed in their medical records were eligible. Participants who agreed to participate in the study voluntarily signed the informed consent form. Exclusion criteria included presence of lower-limb amputations (at any level), active ulcers, and neurological or psychiatric disordersDPN presence was assessed using the Brazilian version of the Neuropathy Disability Score (NDS), validated by Moreira and cols. ^(15)^ and recommended by the Brazilian Diabetes Society ^(16)^, after excluding other potential neuropathy causes. NDS shows high sensitivity (89%) and specificity (100%) for evaluating both small and large nerve fibers based on tests of vibratory sensation, thermal sensitivity, superficial pain, and the Achilles reflex. Based on the score, DPN severity was classified as follows: absent (< 3 points), mild (3–5 points), moderate (6–8 points), and severe (≥ 9 points). Individuals with NDS ≥ 3 were considered to have DPN ^(15)^. Accordingly, participants were divided into two groups: DPN and non-DPN.

Additionally, the Neuropathic Symptom Score (NSS), Visual Analogue Scale (VAS), and Plantar Protective Sensitivity Test (PPS) were applied to further characterize DPN. NSS evaluated the presence and severity of neuropathic symptoms, classified as absent (0–2 points), mild (3–4 points), moderate (5–6 points), or severe (≥ 7 points) [14]. VAS assessed self-reported pain intensity, categorized as absent (0), mild (1–2 points), moderate (3–7 points), or severe (≥ 8 points) ^(17)^. PPS was assessed using the Semmes–Weinstein 10 g monofilament and categorized according to Feng, Schlosser, and Sumpio ^(18)^ as follows: present (preserved sensitivity), reduced (partial sensitivity at some points), or absent (no perception of the tactile stimulus). All neurological tests and foot examinations were performed bilaterally, in a private setting, by a previously trained research team.

Sociodemographic and lifestyle data were collected using a semi-structured questionnaire administered individually and in a private setting by trained members of the research team. Variables included sex (male or female); age group (≤ 59 years, 60–67 years, or > 67 years); self-reported race/skin color (white/yellow or black/mixed-race/indigenous); marital status (with a partner — married or in a stable union — or without a partner — single, widowed, or divorced); schooling level (0–7 years: illiterate or incomplete middle school; ≥ 8 years: complete middle school, incomplete or complete high school, or higher education); work status (active or retired/inactive); current smoking; nutritional therapy adherence and sedentary lifestyle.

To assess nutritional status, body mass index (BMI) was calculated as weight (kg) divided by height squared (kg/m²). Weight and height data were obtained from medical records. Based on BMI values, patients were classified as underweight/eutrophic (BMI < 25 kg/m²), overweight (BMI ≥ 25 and < 30 kg/m²), or obese (BMI ≥ 30 kg/m²), according to the World Health Organization ^(19)^.

Clinical variables were obtained from medical records, including diabetes duration (≤ 10 or > 10 years), glycemic control, and treatment regimen (use of oral antidiabetic drugs and/or insulin therapy). Glycemic control was considered adequate when HbA1c < 7.0%, according to the American Diabetes Association ^(20)^. Presence of comorbidities and complications was investigated, including systemic arterial hypertension, dyslipidemia, myocardial infarction (MI), ischemic stroke, peripheral arterial disease (PAD), retinopathy, and diabetic kidney disease (DKD).

Statistical analyses were conducted using Stata 16.1 (StataCorp LLC, College Station, TX, USA) and RStudio 2024.04.2 (R Foundation for Statistical Computing, Vienna, Austria). First, a univariate analysis was conducted to describe the absolute and relative frequencies of the explanatory variables in relation to the outcome (DPN presence). Chi-squared tests assessed differences in distributions. For laboratory parameters, data non-normality was verified by Shapiro–Wilk test. As the variables did not follow a normal distribution, results were expressed as medians and minimum–maximum values, and comparisons between groups were performed using the Mann–Whitney U test.

Next, a Poisson’s multiple generalized linear model (GLM) with a logarithmic link function and robust variance was fitted to model DPN occurrence as a function of potential predictors. The multivariate analysis only included variables with a *p* < 0.20 in the univariate analysis. Notably, laboratory parameters were excluded from the model due to vast missing information. Variable selection followed the stepwise forward method, and the Akaike Information Criterion (AIC) evaluated the impact of including each predictor on the final model. Variables with p-value < 0.05 were considered significantly associated with the outcome.

Magnitude of associations was estimated using prevalence ratio (PR) and its corresponding 95% CI in both univariate and multivariate analyses. This measure was adopted to avoid overestimating associations in conditions of high outcome prevalence, as recommended by Tamhane and cols. ^(21)^ for cross-sectional studies.

This study was approved by the Human Research Ethics Committee of the Federal University of Rondonópolis, Mato Grosso, Brazil (CAAE no. 47720421.6.0000.0126). All participants provided written informed consent prior to participation.

## RESULTS

Of the 285 patients initially evaluated, nine were excluded: seven due to recent or previous amputation, one due to cognitive impairment, and one due to a neurodegenerative disorder. Our final sample therefore comprised 276 patients.

Of these, 178 were diagnosed with DPN corresponding to a 64.5% prevalence (95% CI, 58.9%–70.1%). **Table 1** presents the distribution of participants according to DPN status, sociodemographic variables, nutritional status, and lifestyle habits. Most DPN patients were female (59.0%), aged over 67 years (37.7%), and those who self-identified as Black, Mixed-race, or Indigenous (71.9%). Most participants reported having a partner (59.6%), tertiary education (61.2%), and being retired or unemployed (81.5%). A total of 77 patients (43.3%) had obesity, and most reported adherence to nutritional guidance (78.1%). Sedentary lifestyle exhibited high frequency (85.4%) and 8.4% reported smoking.

**Table 1.** Participants distribution according to DPN and sociodemographic variables, nutritional status, and lifestyle habits

Variable	DPN				p	PR	95% CI
	No		Yes				
	n	%	n	%			
Sex							
Female	74	75.5	105	59.0	0.006*	Ref.	-
Male	24	24.5	73	41.0		1.28	1.08 - 1.52
Age category (years)^a^							
≤ 59	47	48.0	54	30.3	0.009*	Ref.	-
59 -| 67	28	28.6	57	32.0		1.25	0.99 - 1.59
> 67	23	23.4	67	37.7		1.39	1.12 - 1.73
Race/color							
White/yellow	35	35.7	50	28.1	0.189	Ref.	-
Black/mixed-race/indigenous	63	64.3	128	71.9		1.14	0.93 - 1.40
Marital status							
Without partner	48	49.0	72	40.4		1.13	0.94 - 1.36
With partner	50	51.0	106	59.6	0.171	Ref.	-
Schooling years							
0-7	51	52.0	69	38.8	0.033*	1.21	1.01 - 1.46
≥8	47	48.0	109	61.2		Ref.	-
Employment status							
Retired or inactive	63	64.3	145	81.5	0.002*	1.44	1.11 - 1.87
Active	35	35.7	33	18.5		Ref.	-
Nutritional status^b^							
Underweight or normal weight	16	16.3	35	19.6	0.791	Ref.	-
Overweight	38	38.8	66	37.1		0.92	0.73 - 1.17
Obesity	44	44.9	77	43.3		0.93	0.74 - 1.17
Nutritional therapy							
No	25	25.5	39	21.9	0.498	0.93	0.75 - 1.16
Yes	73	74.5	139	78.1		Ref.	-
Sedentary lifestyle							
No	25	25.5	26	14.6	0.026*	Ref.	-
Yes	73	74.5	152	85.4		1.33	1.00 - 1.76
Smoking							
No	85	89.5	163	91.6	0.567	Ref.	-
Yes	10	10.5	15	8.4		0.92	0.65 - 1.27
Don't know/didn't answer	3	-	-	-		-	-

DPN: diabetic peripheral neuropathy; PR: prevalence ratio; 95% CI: 95% confidence interval.

* Significant at p < 0.05. Chi-squared test.

a Defined according to tertile distribution.

b Defined from Body Mass Index calculation.

Compared with non-DPN individuals, DPN patients differed significantly by sex (p = 0.006), age group (p = 0.009), schooling level (p = 0.033), employment status (p = 0.002), and sedentary lifestyle (p = 0.026) (**Table 1**).

**Figure 1.**
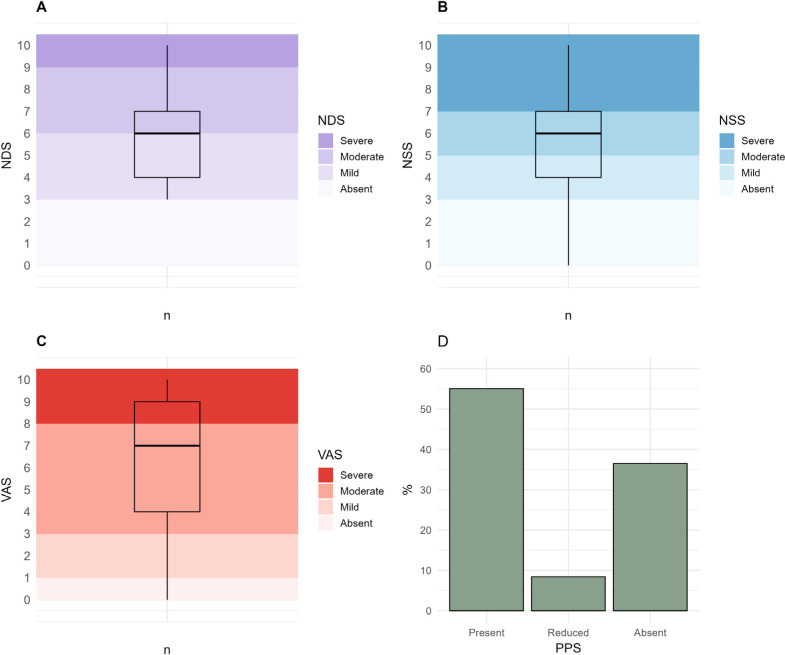
Participants distribution according to DPN characteristics. (**A**) Neuropathy Disability Score (NDS); (**B**) Neuropathy Symptom Score (NSS); (**C**) Visual Analogue Scale (VAS); and (**D**) Plantar Protective Sensation (PPS). Panels A, B, and C are expressed as median values.

Regarding neuropathy characteristics, 158 patients (88.7%) reported experiencing symptoms, and 151 (84.8%) self-reported pain. As shown in **Figure 1**, the study sample exhibited a moderate degree of neuropathy, with moderately intense symptoms and neuropathic pain. Protective plantar sensitivity was absent in over one-third of DPN patients (n = 65; 36.5%).

**Table 2** presents the distribution of participants according to DPN status and clinical variables. Most DPN patients had a diabetes duration longer than 10 years (52.3%), were using insulin (54.5%), and exhibited poor glycemic control (78.9%). Comorbidities presented high prevalence, particularly systemic arterial hypertension (81.5%) and dyslipidemia (82.0%). Among microvascular complications, 73 patients (41.0%) exhibited retinopathy, whereas MI was the most frequent macrovascular complication, occurring in 29 DPN patients (16.3%).

**Table 2.** Participants distribution according to DPN and clinical variables

Variable	NPD				p	PR	95% CI
	No		Yes				
	n	%	n	%			
DM duration (years)							
< 10	58	59.2	85	47.7	0.069	Ref.	
> 10	40	40.8	93	52.3		1.18	0.99 - 1.40
Glicemic control^a^							
No	73	77.7	135	78.9	0.807	1.03	0.82 - 1.28
Yes	21	22.3	36	21.1		Ref.	
Missing data	4	-	7				
Insulin therapy							
No	47	48.0	81	45.5	0.696	Ref.	
Yes	51	52.0	97	54.5		1.03	0.87 - 1.23
Oral antidiabetic drug							
No	4	4.1	12	6.7	0.366	1.17	0.87 - 1.58
Yes	94	95.9	166	93.3		Ref.	
Systemic arterial hypertension							
No	28	28.6	33	18.5	0.055	Ref.	
Yes	70	71.4	145	81.5		1.25	0.97 - 1.60
Dyslipidemia							
No	17	17.3	32	18.0	0.896	Ref.	
Yes	81	82.7	146	82.0		0.98	0.79 - 1.23
Myocardial infarction							
No	93	94.9	149	83.7	0.007*	Ref.	
Yes	5	5.1	29	16.3		1.39	1.17 - 1.64
Ischemic stroke							
No	93	94.9	162	91.0	0.244	Ref.	
Yes	5	5.1	16	9.0		1.20	0.93 - 1.55
Peripheral arterial disease							
No	97	99.0	163	91.6	0.012*	Ref.	
Yes	1	1.0	15	8.4		1.49	1.28-1.75
Retinopathy							
No	69	70.4	105	59.0	0.060	Ref.	
Yes	29	29.6	73	41.0		1.19	1.00 - 1.41
Diabetic kidney disease							
No	87	88.8	140	78.7	0.035*	Ref.	
Yes	11	11.2	38	21.3		1.26	1.05 - 1.51

DPN: diabetic peripheral neuropathy; PR: prevalence ratio; 95% CI: 95% confidence interval.

* Significant at p < 0.05. Chi-squared test.

a Adequate glycemic control defined as HbA1c <7%.

In comparing patients with and without DPN, statistically significant differences were observed for MI (p = 0.007), PAD (p = 0.012), and DKD (p = 0.035). Regarding laboratory parameters, DPN patients presented higher serum creatinine levels (**Table 3**).

**Table 3.** Comparison of laboratory parameters between non-DPN and DPN participants

Parameter	DPN		p
	No	Yes	
FBG (mg/dL) (n=258)	148 (127–196)	159 (120.5–218.5)	0.498
HbA1c (%) (n=265)	8.4 (7.3–10.2)	8.4 (7.2–10.2)	0.953
TC (mg/dL) (n=243)	169.5 (140.5–198.7)	174.0 (139.5–206.9)	0.391
LDL (mg/dL) (n=259)	88.5 (64.59–117.4)	86.2 (60.75–123.0)	0.842
Creatinine (mg/dL) (n=263)	0.8 (0.7–0.99)	0.97 (0.79–1.3)	0.001*
Vitamin B12 (pg/mL) (n=182)	399.0 (336.0–515.0)	456.4 (292.0–658.3)	0.460

DPN: diabetic peripheral neuropathy; FBG: fasting blood glucose; HbA1c: glycated hemoglobin; TC: total cholesterol; LDL: low-density lipoprotein. Results expressed as median (25th–75th percentiles).

* Significant at p < 0.05. Mann–Whitney U test.

**Table 4** presents the variables that remained significantly associated with DPN after adjusting the final multivariate model. DPN prevalence was 32% higher among male than among female patients (PR = 1.32; 95% CI, 1.12–1.56) and 43% higher among retired or inactive participants compared with those active in the labor market (PR = 1.43; 95% CI, 1.11–1.85).

**Table 4.** DPN-associated factors among individuals treated at a reference center. Midwest, Brazil

Variable	p	Adjusted^a^ PR	95% CI
Male sex	0.001*	1.32	1.12 - 1.56
Retired or inactive	0.006*	1.43	1.11 - 1.85
Sedentary lifestyle	0.034*	1.34	1.02 - 1.76
DM duration > 10 years	0.021*	1.22	1.03 - 1.44
Peripheral arterial disease	0.003*	1.25	1.08 - 1.46
Myocardial infarction	0.037*	1.21	1.01 - 1.45

DPN: Diabetic Peripheral Neuropathy. PR: Prevalence ratio. 95% CI: 95% confidence interval.

* Significant at p < 0.05. Poisson Regression.

a Adjusted for: sex, employment status, sedentary lifestyle, duration of diabetes, peripheral arterial disease, and history of myocardial infarction.

Moreover, sedentary individuals had a 34% higher DPN prevalence than physically active ones (PR = 1.34; 95% CI, 1.02–1.76). Additional factors associated with DPN included a diabetes duration longer than 10 years (PR = 1.22; 95% CI, 1.03–1.44), presence of PAD (PR = 1.25; 95% CI, 1.08–1.46), and a history of MI (PR = 1.21; 95% CI, 1.01–1.45).

## DISCUSSION

Diabetic Peripheral neuropathy (DPN) is a serious diabetes complication, contributing to lower-limb amputations, disabling neuropathic pain, and increased mortality ^(22-24)^. Herein, were identified several independent factors associated with this condition, including male sex, retirement or inactivity, longer duration of diabetes, sedentary lifestyle, PAD, and a history of MI. These findings highlight the multifactorial nature of DPN in T2DM patients treated at a referral center in Midwest Brazil.

DPN prevalence was 64.5% (95% CI, 58.9%–70.1%), higher than the prevalence reported in a recent systematic review and meta-analysis which estimated a 46.5% rate (95% CI, 38.0%–55.0%) in Latin America and the Caribbean ^(25)^. Referral bias may partially explain this higher rate, as patients with more severe or complex complications are more likely to receive care at referral centers. Additional contributing factors may include the older age and longer diabetes duration observed among the study participants.

Regarding disease characteristics, 88.7% of patients reported DPN symptoms, indicating a moderate degree of neuropathy. Of the total sample, 84.8% reported pain (**Figure 1**), a frequency higher than that reported by Li and cols. ^(26)^ who found that 57.2% of patients experience painful DPN. In literature, painful DPN is associated with depression ^(27)^, reduced quality of life ^(28,29)^, a greater number of comorbidities, and increased healthcare costs ^(30)^.

In the present study, male sex was significantly associated with a higher DPN prevalence (PR = 1.32; 95% CI, 1.12–1.56), a finding consistent with previous reports ^(31-33)^. Similarly, Aaberg and cols. ^(34)^ observed that men with diabetes developed neuropathic complications at an earlier age (approximately 63 years) than their female counterparts (around 67 years). This finding is further supported by Abraham and cols. ^(35)^, who reported more severe electrophysiological abnormalities in men with diabetes, regardless of the degree of polyneuropathy.

As for sex-related differences, women tend to experience more intense neuropathic symptoms, particularly pain ^(35)^, which is also the most common reason for seeking medical care ^(36)^. Taken together, these findings underscore the importance of considering sex-specific factors in DPN evaluation and management. Targeted screening and preventive strategies should prioritize older male patients with diabetes to delay the onset of neuropathic complications and reduce associated morbidity.

In our study, DPN patients were older (> 67 years) and had a longer diabetes duration (>10 years). Longer T2DM duration is associated with prolonged exposure to chronic hyperglycemia, which intensifies its deleterious effects on multiple organ systems ^(37)^. Consistent with previous studies ^(2,8,11,38-40)^, were observed a significant association between longer diabetes duration and DPN (PR = 1.22; 95% CI, 1.03–1.44). Although a nonmodifiable risk factor, diabetes duration remains an essential indicator for early detection and timely management of DPN ^(41)^.

No significant association was observed between insulin therapy or glycemic control and DPN presence. Nonetheless, 54.5% of patients were using insulin, despite 78.9% achieving inadequate glycemic control. Insulin therapy is often regarded as a marker of long-standing or difficult-to-control T2DM ^(40)^. Conversely, maintaining optimal glycemic control may help delay DPN progression in T2DM patients, although the evidence supporting this association is stronger in T1DM patients ^(42)^.

DPN prevalence was 43% higher among retired or inactive participants compared with those who were economically active (PR = 1.43; 95% CI, 1.11–1.85). Despite limited evidence regarding the association between DPN and employment or retirement status in literature, this finding may reflect the combined effects of advanced age and longer disease duration. According to the Brazilian social security legislation, retirement typically occurs around the age of 60 years or older ^(43)^, reinforcing the plausibility of this observation ^(8,44)^.

Salih and cols. ^(45)^ reported that patients aged ≥ 60 years were 4.47 times more likely to present DPN than those aged 40 (95% CI, 2.40–8.35). The authors attributed this multifactorial association to the combined effects of prolonged diabetes duration such as chronic hyperglycemia, comorbidities, vascular and physiological changes that come with the aging process.

Sedentary lifestyle remained significantly and independently associated with DPN after multivariate analysis (**Table 4**). Similarly, Abdissa and cols. ^(40)^, in a study conducted at a university hospital in Ethiopia, reported that physically inactive patients with diabetes were 2.02 times more likely to develop DPN than those who were physically active (OR, 2.02; 95% CI, 1.14–3.55). Likewise, Salih and cols. ^(45)^ found that physically inactive individuals with T2DM were 1.69 times more likely to develop DPN compared with physically active (95% CI, 1.14–2.49).

Data from the Vigitel survey ^(1)^ indicated a 32.2% (95% CI: 29.5–34.9) prevalence of physical activity among individuals aged 65 years and older. In contrast, 85.4% of patients with DPN reported a sedentary lifestyle, revealing a markedly higher prevalence in this sample. Similarly, Nolan and cols. ^(46)^, who investigated self-reported physical activity levels in T2DM patients aged 33 to 88 years, also observed that individuals with DPN were less physically active than those without DPN.

Positive impacts have been reported from lifestyles that include physical activities and moderate intensity exercise, as well as those focused on flexibility, balance, and range of motion. Such practices have shown beneficial effects for individuals living with T2DM and DPN, including improvements in metabolic profile and microvascular circulation. These effects are associated with the release of neuroprotective factors that attenuate oxidative stress and promote the increase or regeneration of cutaneous nerve fibers, contributing to the reduce neuropathic pain symptoms and, consequently, to improved quality of life ^(2,3,47-51)^.

Diabetes alone significantly increases the cardiovascular risk (CVR) of patients by approximately two- to fourfold when compared with individuals without the disease ^(52)^. Moreover, recent evidence suggests that DPN presence is associated with an additional CVR elevation ^(53)^. High frequencies of CVR factors were identified: systemic arterial hypertension (81.5%), dyslipidemia (82.0%), and obesity (43.3%). Despite no direct association between these risk factors and DPN, results revealed significant associations with two cardiovascular complications: PAD and previous MI. DPN prevalence was 25% higher among patients with PAD and 21% higher among those with a history of MI compared with individuals without these complications (PR = 1.25; 95% CI: 1.08–1.46 and PR = 1.21; 95% CI: 1.01–1.45, respectively).

Consistent with our findings, Pfannkuche and cols. ^(8)^ revealed an independent association between PAD and DPN (OR = 1.81; 95% CI: 1.07–3.08). This coexistence exacerbates vascular dysfunction, thereby increasing the risk of complications like foot ulcers and amputations ^(54)^. Conversely, a previous nonfatal MI, a major adverse cardiovascular event, represents an additional warning sign regarding the clinical condition of these patients and highlights the need for rigorous management of lipid, blood pressure, and glycemic parameters through both pharmacological therapy and lifestyle changes ^(19,54,55)^.

A previous study conducted in London involving 13,043 patients recorded 399 nonfatal cardiovascular events. After adjusting for confounding factors, DPN was associated with a higher incidence of these events among patients with diabetes, with a hazard ratio (HR) of 1.33 (95% CI: 1.02–1.75) ^(56)^. Similarly, in a systematic review focused on patients newly diagnosed with T2DM, Aikaeli and cols. ^(57)^ reported a prevalence of 7% for MI and rates ranging from 1% to 27% for ischemic heart disease among macrovascular complications. These findings underscore the importance of an early diagnosis of T2DM and its complications, given the often silent progression of the disease and the detrimental impact of delayed diagnosis and treatment on patients’ quality of life.

Study limitations include its cross-sectional design, which precludes causal inference, and the use of convenience sampling, which may limit generalizing our findings to the broader population with diabetes. Additionally, data on lifestyle habits were self-reported, which may have led participants to provide socially desirable answers. The high DPN prevalence observed in the study sample may be partially explained by referral bias, as a greater number of patients with complications are typically referred to specialized services. Another important limitation concerns the considerable missing information in medical records regarding laboratory parameters, which prevented a more detailed analysis of their association with DPN and underscored the need for improved documentation of patient clinical data.

As for strengths, we highlight its contribution to expanding knowledge on factors associated with DPN in a municipality in Midwest Brazil, where few investigations on this topic have been conducted. Moreover, the present results may enhance national understanding of diabetic peripheral neuropathy, contributing to public policies and health education in the comprehensive care of Brazilian patients.

## CONCLUSION

DPN was highly prevalent in the study sample, highlighting the urgent need for systematic screening programs focused on early detection and timely initiation of appropriate treatment, as recommended by international reference institutes. Investing in diagnostic equipment and professional training for implementing screening tests, particularly in primary care settings where most patients with T2DM are initially diagnosed, could substantially improve early DPN identification.

Regarding risk factors, greater attention should be directed toward male and older individuals, those with long-standing diabetes, and patients with a history of cardiovascular disease. Lifestyle modification and strict control of cardiovascular risk factors should be strongly encouraged among DPN patients. In this regard, educational initiatives focusing on T2DM management and foot care can help minimize the risk of major complications. Moreover, conducting longitudinal studies can help guide more effective prevention and management strategies of diabetic peripheral neuropathy.

## Data Availability

datasets related to this article will be available upon request to the corresponding author.

## References

[1] Brasil. Ministério da Saúde (2023). Secretaria de Vigilância em Saúde e Ambiente. Departamento
de Análise Epidemiológica e Vigilância de
Doenças Não Transmissíveis. Vigitel Brasil 2023:
vigilância de fatores de risco e proteção para
doenças crônicas nas capitais dos 26 estados brasileiros e no
Distrito Federal em 2023 [recurso eletrônico].

[2] Pop-Busui R, Boulton AJM, Feldman EL, Bril V, Freeman R, Malik RA (2017). Diabetic neuropathy: a position statement by the American
Diabetes Association. Diabetes Care.

[3] Elafros MA, Kellen C, Nix LM, Callaghan BC (2022). Towards prevention of diabetic peripheral neuropathy: clinical
presentation, pathogenesis, and new treatments. Lancet Neurol..

[4] Feldman EL, Callaghan BC, Pop-Busui R, Zochodne DW, Wright DE, Bennett DL (2019). Diabetic neuropathy. Nat Rev Dis Primers..

[5] Lu B, Hu J, Wen J, Zhang Z, Zhou L, Li Y (2013). Determination of peripheral neuropathy prevalence and associated
factors in Chinese subjects with diabetes and pre-diabetes – Shanghai
diabetic neuropathy epidemiology and molecular genetics study
(SH-DREAMS). PLoS One.

[6] Monteiro-Soares M, Santos JV (2022). Diabetes foot-related complications.

[7] Lu Y, Xing P, Cai X, Luo D, Xu G, Zhang L (2020). Prevalence and risk factors for diabetic peripheral neuropathy in
type 2 diabetic patients from 14 countries: estimates of the INTERPRET-DD
study. Front Public Health.

[8] Pfannkuche A, Alhajjar A, Moghadam S, Hamel J, Strom A, Ziegler D (2020). Prevalence and risk factors of diabetic peripheral neuropathy in
a diabetics cohort: register initiative “Diabetes and
Nerves.”. Endocr Metab Sci..

[9] ElSayed NA, Aleppo G, Aroda VR, Bannuru RR, Brown FM, Bruemmer D (2023). Retinopathy, neuropathy, and foot care: Standards of Care in
Diabetes – 2023. Diabetes Care.

[10] Dutra LMA, Araújo LMS, Ferreira C, de Carvalho RBN, Santos ICRV (2018). Assessment of ulceration risk in diabetic
individuals. Rev Bras Enferm..

[11] Reis IFA, Rocha FC, Lima AEO, Pereira LMC, Oliveira GF, Fernandes BKC (2021). Fatores preditivos da neuropatia diabética em idosos
atendidos na atenção primária. Rev Enferm Refer..

[12] Muzy J, Campos MR, Emmerick ICM, Silva RS, Schramm JMA (2021). Prevalência de diabetes mellitus e suas
complicações e caracterização das lacunas na
atenção à saúde a partir da
triangulação de pesquisas. Cad Saude Publica..

[13] Cheng Y, Cao W, Zhang J, Wang J, Liu X, Wu Q, Lin Q (2022). Determinants of diabetic peripheral neuropathy and their clinical
significance: a retrospective cohort study. Front Endocrinol (Lausanne).

[14] Sun J, Wang Y, Zhang X, Zhu S, He H (2020). Prevalence of peripheral neuropathy in patients with diabetes: A
systematic review and meta-analysis. Prim Care Diabetes.

[15] Moreira RO, Castro AP, Papelbaum M, Appolinário JC, Ellinger VC, Coutinho WF (2005). Tradução para o português e
avaliação da confiabilidade de uma escala para
diagnóstico da polineuropatia distal diabética. Arq Bras Endocrinol Metab..

[16] Rolim L, Santos AL, Oliveira JEP, Milech A (2023). Diagnóstico e tratamento da neuropatia periférica
diabética. Diretriz Oficial da Sociedade Brasileira de Diabetes.

[17] Cruccu G, Anand P, Attal N, Garcia-Larrea L, Haanpää M, Jørum E (2004). EFNS guidelines on neuropathic pain assessment. Eur J Neurol..

[18] Feng Y, Schlösser FJ, Sumpio BE (2009). The Semmes Weinstein monofilament examination as a screening tool
for diabetic peripheral neuropathy. J Vasc Surg..

[19] World Health Organization (WHO) (2000). Obesity: preventing and managing the global epidemic: report of a WHO
consultation.

[20] American Diabetes Association (2025). Standards of care in diabetes—2025. Diabetes Care.

[21] Tamhane AR, Westfall AO, Burkholder GA, Cutter GR (2016). Prevalence odds ratio versus prevalence ratio: choice comes with
consequences. Stat Med..

[22] Sloan G, Selvarajah D, Tesfaye S (2021). Pathogenesis, diagnosis and clinical management of diabetic
sensorimotor peripheral neuropathy. Nat Rev Endocrinol..

[23] Ezzatvar Y, García-Hermoso A (2023). Global estimates of diabetes-related amputations incidence in
2010–2020: a systematic review and meta-analysis. Diabetes Res Clin Pract..

[24] Vági OE, Kolossváry E, Zsóri KS, Fekete BC, Kempler P, Lengyel C (2023). The association between distal symmetric polyneuropathy in
diabetes with all-cause mortality: a meta-analysis. Front Endocrinol..

[25] Yovera-Aldana M, Velásquez-Rimachi V, Huerta-Rosario A, Palomino R, Maticorena-Quevedo J, Diez-Canseco F (2021). Prevalence and incidence of diabetic peripheral neuropathy in
Latin America and the Caribbean: a systematic review and
meta-analysis. PLoS One.

[26] Li C, Yang G, Feng Z, Wan L, Wang Z, Wang Y (2023). Prevalence of painful diabetic peripheral neuropathy in type 2
diabetes mellitus and diabetic peripheral neuropathy: a nationwide
cross-sectional study in mainland China. Diabetes Res Clin Pract..

[27] D’Amato C, Morganti R, Greco C, Di Gennaro F, Marfia GA, Giacomelli A (2016). Diabetic peripheral neuropathic pain is a stronger predictor of
depression than other diabetic complications and
comorbidities. Diabetes Vasc Dis Res..

[28] Ernandes RDC, Tavares DMS, Rodrigues RCM, Nogueira GS, Pimenta CJL (2020). Impact of diabetic neuropathy on quality of life and postural
balance in Brazilian older adults. Acta Ortop Bras..

[29] Borbjerg MK, Dalsgaard EM, Andersen ST, Andersen H, Jørgensen ME, Nielsen JS (2025). Understanding the impact of diabetic peripheral neuropathy and
neuropathic pain on quality of life and mental health in 6,960 people with
diabetes. Diabetes Care.

[30] Ebata-Kogure N, Kubo T, Masuda H, Matsushita K, Onishi Y, Takeuchi M (2017). Clinical and economic burdens experienced by patients with
painful diabetic peripheral neuropathy: an observational study using a
Japanese claims database. PLoS One.

[31] Booya F, Bandarian F, Larijani B, Pajouhi M, Nooraei M, Lotfi J (2005). Potential risk factors for diabetic neuropathy: a case control
study. BMC Neurol..

[32] Gogia S, Rao CR (2017). Prevalence and risk factors for peripheral neuropathy among type
2 diabetes mellitus patients at a tertiary care hospital in coastal
Karnataka. Indian J Endocrinol Metab..

[33] Jaiswal M, Divers J, Dabelea D, Isom S, Bell RA, Martin CL (2017). Prevalence of and risk factors for diabetic peripheral neuropathy
in youth with type 1 and type 2 diabetes: SEARCH for Diabetes in Youth
Study. Diabetes Care.

[34] Aaberg ML, Burch DM, Hud ZR, Zacharias MP (2008). Gender differences in the onset of diabetic
neuropathy. J Diabetes Complications.

[35] Abraham A, Barnett C, Katzberg HD, Lovblom LE, Perkins BA, Bril V (2018). Sex differences in neuropathic pain intensity in
diabetes. J Neurol Sci..

[36] Tesfaye S, Boulton AJM, Dyck PJ, Freeman R, Horowitz M, Kempler P (2011). Painful diabetic peripheral neuropathy: consensus recommendations
on diagnosis, assessment and management. Diabetes Metab Res Rev..

[37] Vitale M, Gualdani E, Morieri ML, Bianchi C, Bonora E, Cavalot F (2024). Association between age at diagnosis and all-cause mortality in
type 2 diabetes: the renal insufficiency and cardiovascular events (RIACE)
Italian multicenter study. Acta Diabetol..

[38] Cardoso CRL, Salles GF (2008). Predictors of development and progression of microvascular
complications in a cohort of Brazilian type 2 diabetic
patients. J Diabetes Complications.

[39] Reis de Matos M, Zanella MT, Brandão AP, Campana EMG, Gomes MB (2020). Distal symmetric and cardiovascular autonomic neuropathies in
Brazilian individuals with type 2 diabetes followed in a primary health care
unit: a cross-sectional study. Int J Environ Res Public Health.

[40] Abdissa D, Hamba N, Nigussie D, Adema S (2020). Prevalence and determinants of peripheral neuropathy among type 2
adult diabetes patients attending Jimma University Medical Center, southwest
Ethiopia, 2019: an institutional-based cross-sectional study. J Diabetes Res..

[41] Wang DD, Bakhotmah BA, Hu FB, Alzahrani HA (2014). Prevalence and correlates of diabetic peripheral neuropathy in a
Saudi Arabic population: a cross-sectional study. PLoS One.

[42] Callaghan BC, Price RS, Feldman EL (2012). Enhanced glucose control for preventing and treating diabetic
neuropathy. Cochrane Database Syst Rev..

[43] Brasil. Ministério da Previdência Social (2024). Instituto Nacional do Seguro Social. Regras de Aposentadorias.

[44] Andersen ST, Witte DR, Dalsgaard EM, Andersen H, Nawroth P, Fleming T (2018). Risk factors for incident diabetic polyneuropathy in a cohort
with screen-detected type 2 diabetes followed for 13 years:
ADDITION-Denmark. Diabetes Care.

[45] Salih MH, Woredekal AT, Tadesse F, Abate A, Yimam B, Mohammed S (2024). Peripheral neuropathy and associated factors among type 2
diabetic patients attending referral hospitals in the Amhara region: a
multi-center cross-sectional study in Ethiopia. Sci Rep..

[46] Nolan RC, Dwyer T, Eser P, Kingwell BA, Jones G, Green DJ (2016). Self-reported physical activity using the International Physical
Activity Questionnaire (IPAQ) in Australian adults with type 2 diabetes,
with and without peripheral neuropathy. Can J Diabetes.

[47] Callaghan BC, Gao L, Li Y, Zhou X, Reynolds E, Banerjee M (2021). Dietary weight loss in people with severe obesity stabilizes
neuropathy and improves symptomatology. Obesity (Silver Spring).

[48] Kluding PM, Pasnoor M, Singh R, Jernigan S, Farmer K, Rucker J (2012). The effect of exercise on neuropathic symptoms, nerve function,
and cutaneous innervation in people with diabetic peripheral
neuropathy. J Diabetes Complications.

[49] The Look-AHEAD Research Group (2017). Effects of a long-term lifestyle modification programme on
peripheral neuropathy in overweight or obese adults with type 2 diabetes:
the Look-AHEAD study. Diabetologia.

[50] O’Brien PD, Hinder LM, Callaghan BC, Feldman EL (2017). Neurological consequences of obesity. Lancet Neurol..

[51] Silva Júnior WS, Foss-Freitas MC, Tannus LRM, Oliveira JEP, Gomes MB (2023). Atividade física e exercício no pré-diabetes
e DM2. Diretriz Oficial da Sociedade Brasileira de Diabetes.

[52] Dal Canto E, Ceriello A, Rydén L, Ferrini M, Hansen TB, Schnell O (2019). Diabetes as a cardiovascular risk factor: an overview of global
trends of macro and microvascular complications. Eur J Prev Cardiol..

[53] Schmidt AM (2019). Diabetes mellitus and cardiovascular disease: emerging
therapeutic approaches. Arterioscler Thromb Vasc Biol..

[54] Fitridge R, Hinchliffe RJ, Forsythe RO, Apelqvist J, Boyko EJ, Mills JL (2024). The intersocietal IWGDF, ESVS, SVS guidelines on peripheral
artery disease in people with diabetes and a foot ulcer. Diabetes Metab Res Rev..

[55] Izar M, Lottenberg AM, Fonseca FAH, Sposito AC, Bertoluci MC, Schaan BD (2023). Manejo do risco cardiovascular: dislipidemia. Diretriz Oficial da Sociedade Brasileira de Diabetes.

[56] Brownrigg JR, de Lusignan S, McGovern A, Hughes C, Thompson MM, Ray KK (2014). Peripheral neuropathy and the risk of cardiovascular events in
type 2 diabetes mellitus. Heart.

[57] Aikaeli F, Njim T, Gissing S, Moyo F, Alam U, Mfinanga SG (2022). Prevalence of microvascular and macrovascular complications of
diabetes in newly diagnosed type 2 diabetes in low-and-middle-income
countries: A systematic review and meta-analysis. PLOS Glob Public Health.

